# The Joking Clergyman

**Published:** 1878-11

**Authors:** 


### THE JOKING CLERGYMAN.

REV. DR. BYLES was the most original compound of religion and mirth, conspicuous in the latter part of the last century in New England. With a good heart, a mind of stable principles, and a decent reverence for his holy office, he nevertheless possessed a buoyant and genial flow of spirits, constantly running over with puns or witty conceits. He maintained his connection with the Hollis street church for forty-three years. He was a hale yet aged man when the Revolutionary war began, and in his political predilections leaned towards the royal side.

In May, 1777, it was deemed necessary to arrest him as a Tory. He was condemned to be placed on board a guard ship and sent to England. Subsequently the sentence was changed to confinement in his house. A sentinel was kept before his door day and night, whom he was wont to call his *observ-a-tory.* At the last, the vigilance of the board of war relaxed, and the sentinel disappeared; after a while he was replaced, and after a little removed altogether. The Doctor used pleasantly to remark that he had been "guarded, regarded and disregarded." Once the Doctor tried to have the sentinel let him go after some milk for his family; but he was firm, and would not; he then argued the case with the honest but simple fellow, and actually induced him to go after the milk while he, the Doctor, kept guard over himself! The neighbors were filled with wonderment to see their pastor walking in measured strides before his own door with the sentinel's gun at his shoulder, and when the story got abroad, it furnished food for town gossip and merriment for several days.

The Doctor had rather a shrewish wife; so one day he called at the old distillery that used to stand on Lincoln street, and accosted the proprietor thus:

"Do you still?"

"That is my business," replied Mr. Hill, the proprietor.

"Well, then," said the Doctor, "I should like to have you go and still my wife."

He served rather an ungallant trick upon this same good lady at another time. He had some curiosities, which people occasionally called to see. One day two ladies called. Mrs. B. was "in the suds," and begged her husband to shut her in a closet while he exhibited his curiosities. He did so. After exhibiting everything else he said, "Now, ladies, I have reserved my greatest curiosity to the last," and opening the door he exhibited Mrs. B. to the ladies.

There was an unseemly "slough of despond" before his door, in the shape of a quagmire, which he had repeatedly urged the town authorities to remove. At last two of the town officers in a carriage got fairly stuck in it. They whipped the horse, they hawed and geed, but they could not get out. Dr. Byles saw them from his window. He stepped out in the street—"I am delighted, gentlemen," said he rubbing his hands with glee, "to see you *stirring in this matter* at last!" The sore in the ground was healed soon after.

Going along the street one day he found himself in a great crowd near the old North Church.

"What is the matter?" inquired he of a bystander.

"Why, sir, there is a man going to fly from the steeple."

"Poh! poh!" said he, "do you all come here to see a man fly? Why, I have seen a horse-fly."

A learned lady of Boston despatched a note to him on the Great Dark Day, (May 19, 1780,) in the following style:

"Dear Doctor,—How do you account for this darkness?"

His reply was—

"Dear Madam,—I am *as much in the dark* as you are."

Reader! study now, to have a healthful old age, and then, if good, you can afford to be mirthful, like the brave old Dominie

